# Genomic Epidemiology of Antibiotic-Resistant Bacteria Sampled from Metropolitan Wastewater

**DOI:** 10.3390/microorganisms14050961

**Published:** 2026-04-24

**Authors:** Jakobi T. Deslouches, Nathan J. Raabe, Emma G. Mills, Giuseppe Fleres, Nathan R. Wallace, Mohamed H. Yassin, Daria Van Tyne

**Affiliations:** 1 Division of Infectious Diseases, University of Pittsburgh School of Medicine, Pittsburgh, PA 15213, USA; 2 Microbial Genomic Epidemiology Laboratory, Center for Genomic Epidemiology, University of Pittsburgh, Pittsburgh, PA 15260, USA; 3 Department of Epidemiology, School of Public Health, University of Pittsburgh, Pittsburgh, PA 15260, USA

**Keywords:** antibiotic resistance, genomic epidemiology, wastewater surveillance, plasmids, resistance monitoring

## Abstract

Wastewater surveillance is an effective approach for monitoring populations of antibiotic-resistant bacteria and tracking the spread of antimicrobial resistance (AMR) across different settings. In this study, hospital and municipal wastewater were collected monthly for 12 months from multiple locations in the greater Pittsburgh area to quantify the presence of antibiotic-resistant bacteria and investigate their genomic diversity. After quantitative culturing on six different selective media types, a total of 150 isolates were speciated by 16S rRNA sequencing, which revealed diverse pathogenic and non-pathogenic taxa, including *Klebsiella* spp. (n = 28), *Pseudomonas* spp. (n = 20) and *Aeromonas* spp. (n = 37). A subset of isolates (n = 46) underwent whole genome sequencing, which identified several antibiotic resistance genes of clinical concern, such as *bla*_KPC_ (n = 17), *bla*_NDM_ (n = 6) and *bla*_IMP_ (n = 6), and revealed genetic similarities between wastewater isolates and clinical isolates collected from infected patients at a Pittsburgh-area medical center. In addition, analysis of plasmids carried by wastewater isolates revealed closely related plasmids present in isolates from different species and sampling locations. Overall, these findings suggest that both hospital and municipal wastewater act as interconnected reservoirs of antimicrobial resistance. Integrating wastewater surveillance with clinical and genomic data could enable the early detection of emerging resistance threats and support proactive infection-control strategies.

## 1. Introduction

Bacteria are developing resistance to antibiotics at an alarming rate, with many strains exhibiting resistance to multiple classes of antibiotics [1]. These resistant organisms pose a burden to healthcare systems by increasing morbidity, mortality, and healthcare costs [1,2]. Many antibiotic-resistant infections are caused by a group known as ESKAPE pathogens (*Enterococcus* spp., *Staphylococcus aureus*, *Klebsiella pneumoniae*, *Acinetobacter baumannii*, *Pseudomonas aeruginosa*, and *Enterobacter* spp.), which includes pathogens encoding multiple strategies for developing resistance to commonly used antibiotics like beta-lactams, fluoroquinolones, and macrolides [3]. Most ESKAPE pathogens develop resistance through horizontal gene transfer (HGT), which involves the sharing of mobile genetic elements (MGEs) such as plasmids, transposons, prophages, and insertion sequences [4,5,6]. Multidrug-resistant ESKAPE pathogens cause hospital-acquired infections which are often difficult to treat and have high mortality rates [3,7].

Microbial surveillance is the monitoring of microorganisms present in a specific area or region over time. Surveillance of microorganisms can aid in epidemiologic studies, including point-prevalence surveys and outbreak investigations [8]. Wastewater surveillance is a form of microbial surveillance that samples water from a sewer system adjacent to a community and is sometimes more feasible than obtaining biospecimens directly from infected patients [9]. Wastewater surveillance may also capture a larger population of organisms, as it aggregates sampling across an entire municipality or catchment area. Furthermore, wastewater systems harbor high microbial diversity, providing an environment where HGT frequently occurs among bacteria [10]. Wastewater surveillance was used to monitor the prevalence of SARS-CoV-2 during the COVID-19 pandemic, and has been used in the past to monitor the presence and prevalence of antibiotic resistance genes [9,11]. The use of whole genome sequencing (WGS) provides additional opportunities for comparative analyses between antibiotic-resistant bacteria sampled from wastewater and those collected from clinical settings [12].

Here we combined wastewater surveillance and WGS to quantify and characterize antibiotic-resistant bacteria sampled from hospital and municipal wastewater sources in the Pittsburgh area over a 12-month period. We performed quantitative plating, species typing, WGS, and comparative genomics analysis to understand the prevalence and diversity of antibiotic-resistant bacteria sampled from both hospital and municipal environments. We also compared the genomes of antibiotic-resistant bacteria isolated from wastewater with those of clinical isolates collected from a local hospital system through the Enhanced Detection System for Healthcare-Associated Transmission (EDS-HAT) project [13,14], to explore the genomic epidemiology of antibiotic resistance across both hospital and municipal wastewater sites.

## 2. Methods

### 2.1. Wastewater Sampling

Hospital and municipal wastewater samples were collected concurrently and approximately monthly over a 12-month period (September 2023 through August 2024) from four different sampling locations (two hospital sites and two municipal sites) in the Pittsburgh area on a concurrent basis. Hospital and municipal sampling sites were geographically distinct and unconnected from one another. On the day of collection, wastewater samples from each site were serially diluted 10-fold in 1x Phosphate Buffer Saline (PBS) and 10 µL of each dilution was plated onto selective media agars. Selective plates used included MacConkey agar containing 1 µg/mL ciprofloxacin for detection of fluoroquinolone-resistant Enterobacterales, MacConkey agar containing 1 µg/mL cefotaxime for detection of ESBL-producing Enterobacterales, MacConkey agar containing 1 µg/mL meropenem for detection of carbapenem-resistant Enterobacterales, Pseudomonas isolation agar containing 1 µg/mL meropenem for detection of carbapenem-resistant *Pseudomonas* spp., mannitol salt agar containing 4 µg/mL oxacillin for detection of methicillin-resistant *Staphylococcus* spp., and bile esculin azide agar containing 10 ug/mL vancomycin for the detection of vancomycin-resistant *Enterococcus* spp. After overnight incubation at 37 °C, colony-forming units (CFU) per mL were calculated and recorded for each collection site and timepoint by plate type.

### 2.2. 16S rRNA Sequencing

Seven different sampling time points were selected for isolation of individual colonies from selective media plates. Between 1–3 individual colonies with different morphologies (150 isolates total) were picked from selective plates and passaged on Brain Heart Infusion (BHI) agar with no antibiotic selection to create monocultures. Plates were scraped and isolates were cryopreserved in 1 mL BHI media + 50% glycerol and stored at −80 °C. 16S rRNA typing was performed by Azenta Life Sciences (Burlington, MA, USA) using PCR assays to type the V1 to V9 regions of the bacterial 16S rRNA gene.

### 2.3. Whole Genome Sequencing (WGS) and Comparative Genomics Analysis

A total of 46 antibiotic-resistant wastewater isolates belonging to clinically relevant bacterial species were selected to undergo WGS. DNA from pure overnight cultures grown in tryptic soy broth (TSB) media at 37 °C shaking at 170 rpm was extracted using a Qiagen DNeasy Blood and Tissue Kit (Hilden, Germany) following the manufacturer’s instructions. After quantification using a Qubit fluorimeter (Thermo Fisher Scientific, Waltham, MA, USA), next-generation sequencing libraries were prepared and sequenced using 2 × 150 paired-end reads on an Illumina NextSeq at SeqCenter (Pittsburgh, PA, USA). Kraken2 v2.1.2 was used for initial taxonomic classification of all WGS samples [15]. Reads were assembled into contigs (≥500 bp) using SPAdes v3.15.5 [16], and QUAST v5.2.0 [17] was used to assess the quality of each assembled genome. To verify taxonomic classifications, Kleborate v2.4.1 [18] was used for *Klebsiella* spp. and GTDB-Tk v2.4.1 [19] was used for other genera. Prokka v1.14.5 [20] was used to annotate all genomes, while GTDB-Tk was used to generate a core genome alignment and RAxML v8.2.12 [21] was run using the GTRCAT algorithm with 100 bootstraps to generate a phylogenetic tree for subsequent visualization in iTOL v7.2 [22]. AMRFinderPlus v4.0.22 [23] was used to screen for the presence of antibiotic resistance genes, using the -organism flag for the most closely related species in the database and an identity threshold of ≥90%. The genetic relatedness of wastewater isolate genomes was determined using whole genome single-nucleotide polymorphisms (SNPs) identified via Split Kmer Analysis (SKA) v1.0 [24]. Multilocus sequence types (STs) were identified using the PubMLST database with mlst v2.11 [25,26]. Genomes were also compared to previously collected hospital isolates deposited in NCBI BioProject PRJNA475751 using SKA and a 20 SNP cut-off for clustering of genetically similar isolates.

### 2.4. Long Read Sequencing and Hybrid Assembly

A total of 19 isolates were selected for long-read sequencing on the Oxford Nanopore Technology MinION platform (Oxford, UK). DNA from pure overnight cultures grown in TSB at 37 °C shaking at 170 rpm was extracted using a Qiagen DNeasy Blood and Tissue Kit (Hilden, Germany) following the manufacturer’s instructions. Sequencing libraries were prepared using the SQK-RBK114.24 rapid gDNA Barcoding Kit (Oxford Nanopore Technologies, Oxford, UK) and were sequenced for 72 h using an Oxford Nanopore Technologies (ONT) MinION Mk1C on R10.4.1 flow cells. Basecalling and demultiplexing were performed using Dorado v0.9.6. Hybrid assembly was performed using both short-read (Illumina) and long-read (ONT) data for each isolate with Unicycler v0.5.1 [27], and de novo assemblies were visualized using Bandage v0_8_1 [28]. QUAST v5.2.0 was used to screen for contamination, verify genome sizes, and perform other quality control checks on each hybrid assembly [17].

### 2.5. Plasmid Analysis

Plasmids which met the following inclusion criteria were identified and extracted from hybrid assemblies: circular sequence, ≥10 kb in length, and a plasmid incompatibility group and/or replicon identified by mob_typer v3.1.9 using default parameters [29]. For plasmids where a generic replicon group was assigned (i.e., “rep_cluster”), the corresponding sequence from the mob_typer database was queried using BLASTpv2.17.0 against the PlasmidFinder database [30] using >80% similarity score (% coverage × % identity) to identify the closest replicon type [31]. Using these criteria, a total of 57 plasmid contigs were extracted from hybrid assemblies across 19 antibiotic-resistant wastewater isolates. ABRicate v1.0.1 was used to identify antibiotic resistance genes within plasmids using the Resfinder database v4.0 [32] with ≥80% cutoffs for percent identity and coverage. Pling v2.0.0 [33] was used to quantify plasmid similarity and perform clustering of highly related plasmids into communities (containment distance <0.3) and subcommunities (additionally, DCJ-indel distance ≤4). Clusters of similar plasmids were visualized using Gephi v0.10 [34]. Clustered plasmids were annotated using Prokka v1.14.5 [20] and aligned with Easyfig v2.2.5 to generate multiple sequence alignments [35].

### 2.6. Statistical Analysis

Differences in bacterial abundance between sampling locations were assessed in a pairwise fashion via Mann–Whitney test adjusted for multiple comparisons.

## 3. Results

### 3.1. Population Dynamics of Resistant Organisms over Time

**Figure 1:**
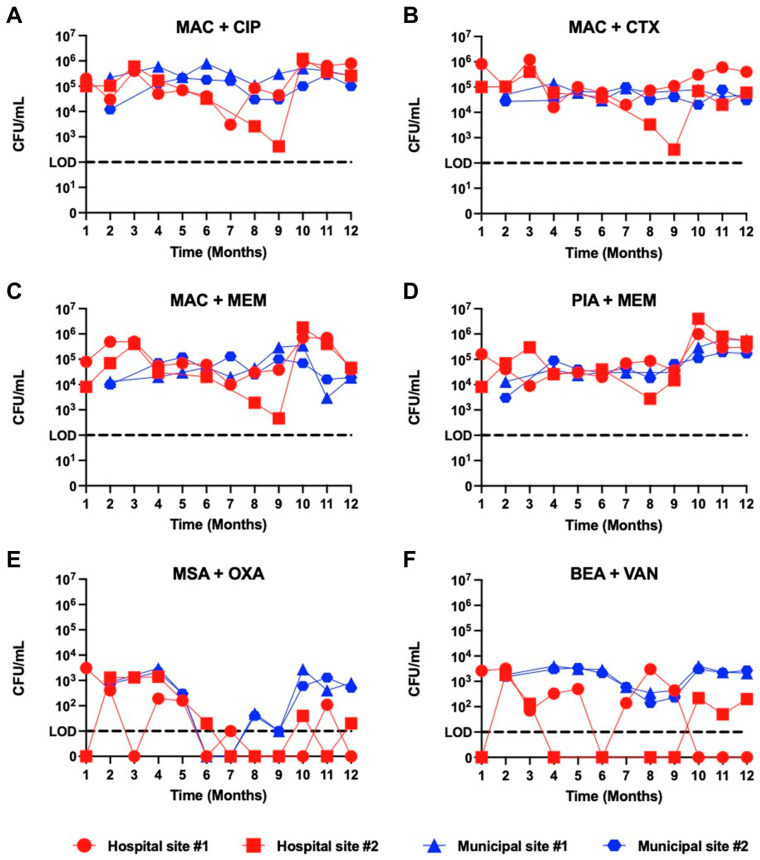
Temporal quantification of resistant organism burdens from hospital and municipal wastewater samples grown on selective media types. Tracking of colony-forming units (CFU) per mL over time for four different wastewater sources sampled over a 12-month period by plating onto six different selective media types, including (**A**) MacConkey agar (MAC) containing 1 µg/mL ciprofloxacin (CIP), (**B**) MAC containing 1 µg/mL cefotaxime (CTX), (**C**) MAC containing 1 µg/mL meropenem (MEM), (**D**) Pseudomonas isolation agar (PIA) containing 1 µg/mL MEM, (**E**) Mannitol salt agar (MSA) containing 4 µg/mL oxacillin (OXA), and (**F**) Bile esculin azide agar (BEA) containing 10ug/mL vancomycin (VAN). Colors and symbol shapes distinguish hospital and municipal sites, and lines connect data points from the same sampling site.

To assess the abundance of antibiotic-resistant bacteria present in hospital and municipal wastewater, we sampled four different sites (two hospital, two municipal) located in the Pittsburgh, Pennsylvania metropolitan area monthly over a one-year period and used selective media plating to quantify resistant organism burdens across each site and over time. Temporal monitoring showed variable dynamics across each wastewater source (Figure 1, Table S1). Overall, plating of wastewater onto MacConkey agar containing ciprofloxacin, cefotaxime, or meropenem, as well as Pseudomonas isolation agar with meropenem, revealed an abundance of growth, with 10^4^–10^5^ colony-forming units (CFU) per mL detected on average year-round. The abundance of growth on these plates from hospital wastewater was more variable month-to-month compared to municipal wastewater, which showed less variability between sampling time points. Plating of wastewater onto mannitol salt agar with oxacillin and bile esculin azide agar with vancomycin yielded far lower CFU/mL burdens compared to the other selective media, with some hospital sampling timepoints showing no growth above the limit of detection. Across all sampling locations and timepoints, plating on Gram-negative–selective media yielded an average of 2.0 × 10^5^ ± 3.1 × 10^4^ CFU/mL on average, whereas plating on Gram-positive–selective media yielded 1.2 × 10^3^ ± 1.6 × 10^2^ CFU/mL on average, representing a nearly 170-fold difference in abundance (Table S1). Additionally, the abundance of organisms from municipal sources that grew on these plates was seasonally variable, with lower abundances apparent in months six through nine. We also compared microbial load on each selective media between sampling locations, and observed modest but significant differences in growth between the two municipal sites on MacConkey agar with ciprofloxacin and on bile esculin azide agar with vancomycin (Figure S1). Overall, these data suggest that antibiotic-resistant organisms were detected at moderate abundance in both hospital and municipal wastewater, and only modest differences in abundance were observed between different sample locations and times.

### 3.2. 16S rRNA Typing of Wastewater Isolates

**Figure 2:**
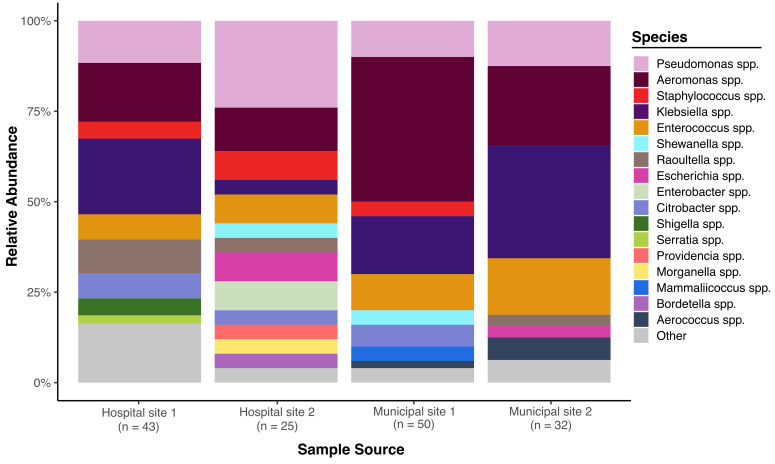
Relative abundance of bacterial genera isolated from hospital and municipal wastewater. Isolated colonies (n = 150 total) collected from wastewater samples plated on selective media were typed via 16S rRNA sequencing. The relative abundance of isolates belonging to each genus from each sampling location is shown.

To assess the taxonomic distribution of organisms that grew on the selective media, we performed 16S rRNA typing on 150 isolates sampled from the four different sites at seven different sampling timepoints. While a wide variety of organisms were identified at each site, the most abundant genera observed across the dataset included *Aeromonas* spp. (n = 37 isolates, 25%), *Klebsiella* spp. (n = 28 isolates, 19%), *Pseudomonas* spp. (n = 20 isolates, 13%), and *Enterococcus* spp. (n = 15 isolates, 10%) (Figure 2, Table S2). These genera were identified at all four sites, while *Staphylococcus* spp., *Raoultella* spp., and *Citrobacter* spp. were identified at three of the four sites. There was no apparent enrichment of any genera at any site, suggesting that bacterial communities across the four locations largely comprised similar taxa.

### 3.3. Genomic Analysis of Wastewater Isolates

**Figure 3:**
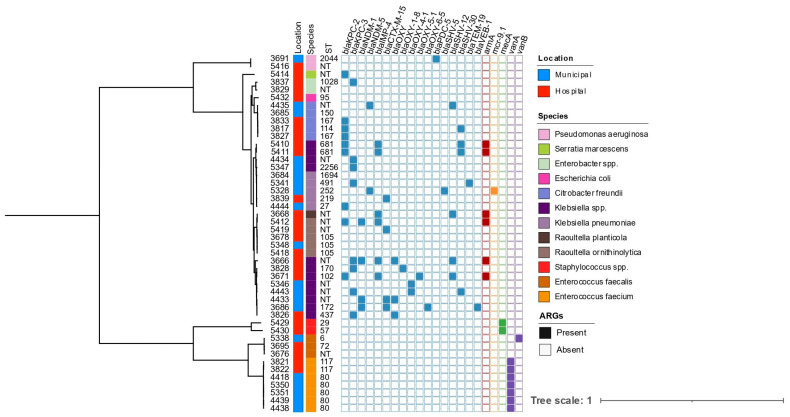
Genetic relatedness and presence of high-risk antimicrobial resistance genes among 46 isolates collected from wastewater sources. The midpoint-rooted phylogenetic tree was constructed using RAxML with 100 bootstraps based on a 120 bacterial marker gene alignment produced by GTDB-Tk. The location where isolate was collected from is shown as municipal (blue) or hospital (red) wastewater sources. Species group are colored as indicated. Multilocus sequence types are noted, those non-typable are indicated as “NT”. Antimicrobial resistance genes were identified using AMRFinderPlus with gene presence noted by a filled square. Genes are color based on expected phenotypic resistance: aminoglycoside (red), β-lactams (light blue), colistin (orange), methicillin (green), and glycopeptide (purple).

The 16S rRNA typing analysis identified several species belonging to the ESKAPE group of pathogens as well as other pathogenic Enterobacterales such as *Citrobacter* spp., *Raoultella* spp., and *Serratia* spp. To investigate the genomic diversity of isolates belonging to these pathogen groups, we performed whole genome sequencing of 46 antibiotic-resistant wastewater isolates on the Illumina platform and constructed a maximum likelihood phylogenetic tree based on an alignment of 120 conserved bacterial core genes (Figure 3). Isolates were broadly divided into Gram-positive (n = 12) and Gram-negative (n = 34) groups. Species identification revealed a diverse population of organisms, with *Klebsiella* spp. (n = 18, 39%), *Enterococcus* spp. (n = 13, 28%), and *Raoultella* spp. (n = 6, 13%) being the most frequently observed (Table S3). Species identification from genomic data largely agreed with 16S typing results, with the exception of isolates within the *Klebsiella pneumoniae* species complex for which WGS showed greater accuracy in species identification.

To assess for the presence of antibiotic resistance genes among sequenced isolates, isolate genomes were queried against the AMRFinderPlus v4.2.7 database. This analysis revealed that most wastewater isolates possessed genes predicted to confer resistance to multiple antibiotic classes. (Figure 3, Table S3). The most frequently observed classes of antibiotic resistance genes included aminoglycosides, β-lactams, and quinolones, with different resistance patterns apparent between the different genera sampled. Of the 34 Gram-negative isolates sequenced, 22 isolates (65%) were carbapenemase-positive, most commonly due to the presence of a *bla*_KPC_ (n = 17, 77%), *bla*_NDM_ (n = 6, 27%), and/or *bla*_IMP-4_ (n = 6, 27%). Seven isolates encoded more than one carbapenemase, with two isolates collected from hospital wastewater carrying three different enzymes. Six isolates from diverse *Klebsiella* (n = 4) and *Raoultella* species (n = 2) encoded the *armA* 16S methyltransferase, which is predicted to confer resistance to all clinically relevant aminoglycosides [36]. Concerningly, these isolates were also carbapenemase-positive. Additionally, of all sequenced isolates, 39% (n = 18) encoded extended-spectrum β-lactamases (ESBLs), including a variety of *bla*_CTX_, *bla*_OXY_, and *bla*_SHV_ enzymes. A single *Klebsiella pneumoniae* isolate encoded the colistin resistance gene *mcr-9.1* [37] in addition to the *bla*_NDM-5_ carbapenemase and the *bla*_CTX-M-15_ ESBL. We identified two *E. faecalis* isolates that grew on vancomycin-supplemented media despite lacking a detectable van operon, suggesting breakthrough growth or loss of the van operon during laboratory propagation prior to WGS. Nonetheless, our results indicate that high-risk antimicrobial resistance genes are not limited to healthcare settings, as they were detected in both municipal and hospital wastewater samples.

### 3.4. Genetic Relatedness of Wastewater and Clinical Isolates

**Figure 4:**
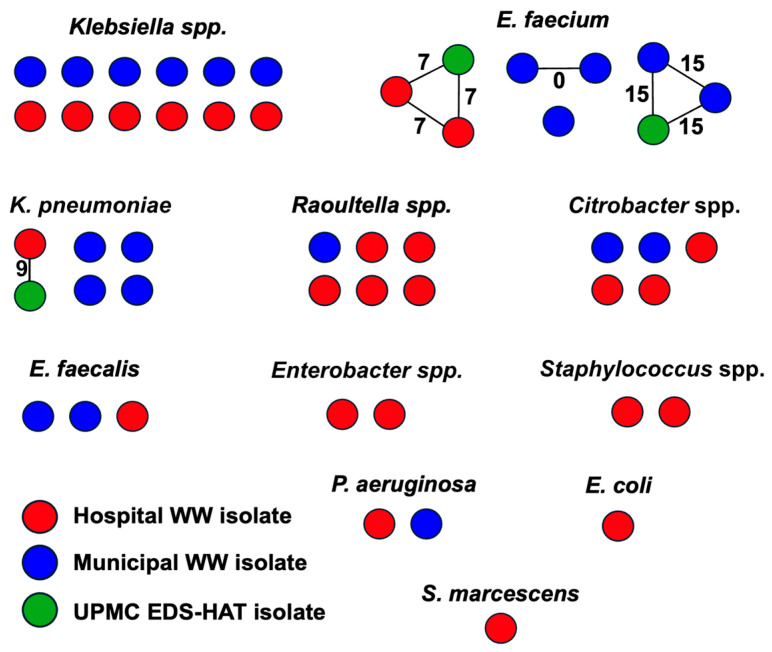
Cluster network plot of genetic relatedness between isolates sampled from wastewater and clinical isolates. Red circles represent hospital wastewater isolates, blue circles represent municipal wastewater isolates, and green circles represent clinical isolates collected from the Enhanced Detection System for Healthcare-Associated Transmission (EDS-HAT) program. Isolates are grouped by species or genus. *Klebsiella* spp. includes non-*K. pneumoniae* isolates. Solid lines connect isolates that are closely related (i.e., ≤15 single-nucleotide polymorphisms, SNPs, apart). SNP distances between clustered isolate pairs are listed next to each line.

To assess the genetic relatedness of antibiotic-resistant wastewater isolates between each other and on a larger epidemiological scale, we quantified the number of single-nucleotide polymorphisms (SNPs) between sequenced isolates sampled from wastewater as well as between wastewater isolates and clinical isolates collected from infected patients at the University of Pittsburgh Medical Center (UPMC) (Figure 4). Three pairs of *E. faecium* isolates sampled from wastewater were found to be closely related to one another (0–15 SNPs apart). These isolates were collected from the same sampling location and same time point, suggesting that they represent strains that were abundant in the water that was sampled. We also identified three clinical isolates (2 *E. faecium* and 1 *K. pneumoniae*) that were closely related to wastewater isolates (7–15 SNPs apart). In two instances, the wastewater isolates were collected from hospital wastewater, although the hospitals where the wastewater was collected differed from those where the patients were sampled. The three *E. faecium* clusters contained isolates belonging to pandemic lineages ST80 and ST117, which are increasing in prevalence both locally and globally [38,39] (Table S3). Taken together, these data suggest that most wastewater isolates in our study were unrelated to one another. However, the instances of genetic relatedness between wastewater and clinical samples demonstrate the potential for spread of resistant organisms between clinical and environmental settings.

### 3.5. Genetically Similar Plasmids Identified in Hospital and Municipal Wastewater Isolates

**Figure 5:**
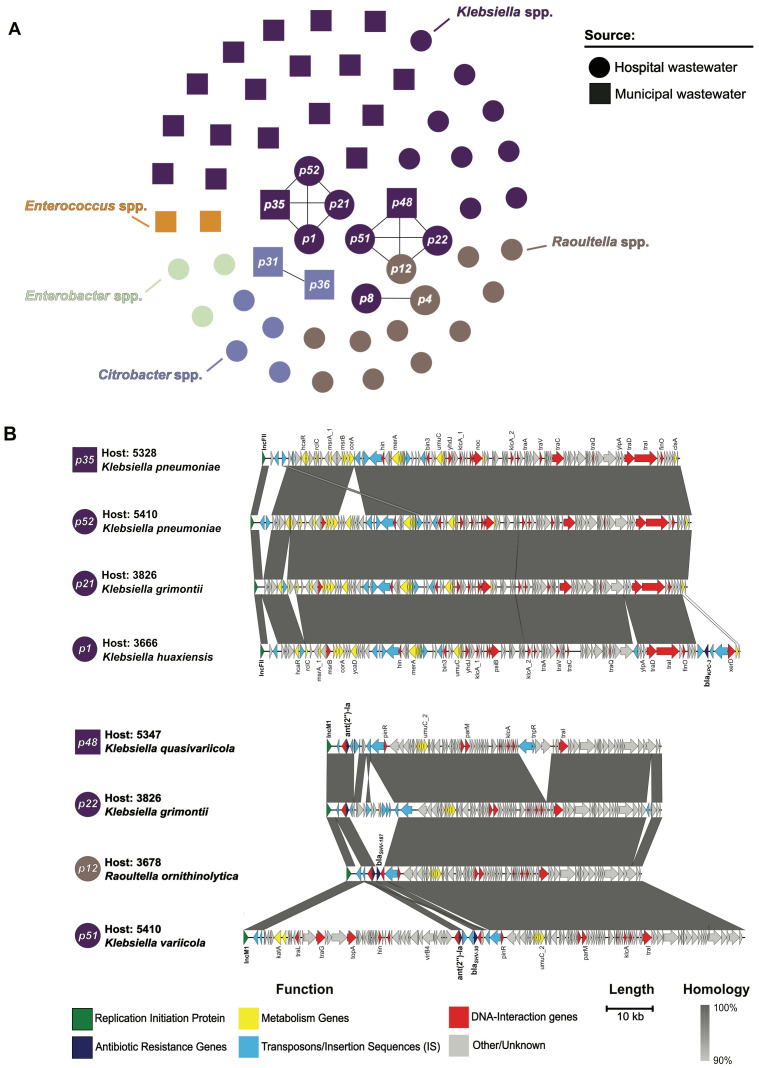
Genetic relatedness of plasmids encoded by antibiotic-resistant wastewater isolates. (**A**) Cluster plot of 57 extracted plasmids from wastewater isolates subjected to MinION sequencing. Plasmids are grouped by species group and genetically similar plasmids (double-cut-and-join value ≤4, containment <0.3 identified by Pling) are connected with lines. (**B**) Multiple sequence alignments of two clusters of genetically similar plasmids. Grey shading indicates genetically similar sequences (≥90% identity, ≥500 bp length). Open reading frames are colored based on gene annotation, and plasmids are labeled as in (**A**).

Of the 46 sequenced wastewater isolates, we selected 19 isolates for MinION sequencing, performed hybrid genome assembly, and extracted a total of 57 circular plasmids greater than 10 kb in length with an identifiable plasmid incompatibility group and/or replicon type from the resulting genomes (Table S4). Among these 57 plasmids we identified 16 distinct incompatibility groups and/or replicon types, most commonly IncFII (n = 27, 47%), IncFIB (n = 13, 23%), IncM1 (n = 4, 7%), and IncU (n = 4, 7%). Many of these plasmids encoded antibiotic resistance genes, some of which confer resistance to multiple classes of antibiotics, namely β-lactams, aminoglycosides, and macrolides. Clustering of plasmid sequences using Pling [33] identified four clusters of genetically similar plasmids with distinct incompatibility/replicon types containing two to four plasmids each: IncFII (n = 4), IncM1 (n = 4), IncU/repFIB/repHI5B (n = 2), and IncX3 (n = 2) (Figure 5A, Table S4). The two largest clusters (IncFII and IncM1) contained plasmids from different species and were recovered from different sampling locations, including both hospital and municipal wastewater sources. Within a cluster, plasmids demonstrated reasonably high homology between shared sequences, but the presence of antibiotic resistance genes varied between plasmids (Figure 5B). Recovery of highly similar plasmids from isolates originating from both hospital and municipal wastewater sources suggests that some plasmids may circulate between these wastewater systems and could transfer antibiotic resistance and other genes between bacteria in these settings.

## 4. Discussion

This study monitored the occurrence of antibiotic-resistant bacteria sampled from hospital and municipal wastewater across the greater Pittsburgh area, and characterized the genomic variability and antibiotic resistance gene content of nearly 50 individual isolates sampled from wastewater. By combining longitudinal sampling, selective culturing, whole-genome sequencing, and comparative analysis of bacterial and plasmid sequences, we captured both the abundance and diversity of antibiotic-resistant taxa, and resolved the genetic contexts of their antimicrobial-resistance determinants. Comparisons with contemporaneous clinical isolates from a hospital in our region revealed closely related isolates that were shared between wastewater and patient populations, suggesting possible overlap between environmental and clinical reservoirs.

Similar to prior studies, we identified a diverse array of antibiotic-resistant bacterial species, most commonly *Aeromonas* spp., *Klebsiella* spp., *Pseudomonas* spp., and *Enterococcus* spp. within both hospital and municipal wastewater settings [40,41,42,43,44,45,46,47]. *Klebsiella* spp., *Pseudomonas* spp., and *Enterococcus* spp. are opportunistic pathogens with antibiotic resistance primarily driven by gene acquisition or chromosomal alterations [48,49,50]. *Aeromonas* spp. are considered environmental and nonpathogenic to humans; however, they can cause opportunistic infections and are intrinsically resistant to carbapenems due to a chromosomally encoded metallo-β-lactamase genes [51]. Previous wastewater surveillance studies using PCR-based methods have reported carbapenemase profiles similar to those observed here, including *bla*_KPC_, *bla*_NDM_, and *bla*_IMP_ variants [42,52,53]. However, by using whole-genome sequencing, here we identified both the presence of these resistance determinants and their distribution across bacterial genera in both hospital and community wastewater. This comparative genomic approach provides greater insight into potential mechanisms driving antimicrobial resistance dissemination within interconnected environmental and healthcare reservoirs. These insights could be useful for developing interventions aimed at eliminating dangerous pathogens from these reservoirs, such as targeted cleaning or replacement of sewer lines and wastewater treatment with ozonation or disinfectants.

To investigate potential connections between environmental and clinical reservoirs of antimicrobial resistance, we compared the genomes of wastewater isolates with each other and with patient isolates collected through a local healthcare-associated transmission surveillance program [13]. This analysis revealed three putative clusters consisting of both wastewater and clinical isolate genomes, suggesting possible overlap between clinical and environmental settings. Notably, the clustered *E. faecium* isolates belonged to globally disseminated lineages ST117 and ST80, both of which are associated with hospital-adapted clades responsible for outbreaks and persistent colonization in healthcare systems [38,53,54]. The detection of these epidemic lineages in wastewater aligns with prior studies showing wastewater as a reservoir for healthcare-associated pathogens [55,56]. Furthermore, *E. faecium* and *K. pneumoniae* are the most frequent causes of hospital outbreaks at our healthcare center [14,57,58], which aligns with our observation of closely related isolates of these species also detected in wastewater. While most isolates in our collection were genetically distinct from one another, the small number of highly related isolates we identified shows the importance of genomic surveillance to monitor the spread of antibiotic-resistant pathogens in environmental and community settings.

Through comparing the sequences of plasmids encoded by the sampled isolates, we identified four clusters of closely related plasmids present in diverse isolates sampled from municipal and hospital wastewater sources. These clusters all contained conjugative plasmids, which can readily transfer between bacterial hosts and facilitate the spread of antimicrobial resistance elements across both hospital and municipal wastewater environments [59,60,61]. Consistent with previous reports, our findings show plasmids as a likely driver of antimicrobial resistance propagation within diverse bacterial populations and environmental reservoirs [59,62]. Notably, we identified two isolates carrying an IncX3 plasmid encoding *bla*_NDM-5_ which were both collected from municipal wastewater. IncX3 plasmids harboring *bla*_NDM-5_ have been widely reported across multiple countries and isolated from various sources including hospitalized patients, agriculture settings, and wastewater [63,64,65,66]. Our findings, in conjunction with other reports [59,60,61,63], highlight wastewater as a potential reservoir for plasmid-mediated dissemination of high-risk resistance genes, showing the need for continued environmental surveillance.

While our approach uncovered key genomic similarities between environmental and clinical antibiotic-resistant bacteria, some limitations should be acknowledged. First, our analysis prioritized sampling depth over breadth, focusing on four specific sewer lines within the greater Pittsburgh area and therefore limiting broader geographic inferences. Second, only a subset of clinically relevant genera underwent whole-genome sequencing, leaving non-clinical genera such as *Aeromonas* and *Shewanella* excluded from downstream analyses despite their recognized roles in antimicrobial resistance dissemination [67,68,69]. In addition, the use of culture-based methods inherently biased our detection toward organisms capable of growth under the conditions used, which overlooked viable but non-culturable, slow-growing, anaerobic, and low-abundance taxa. Metagenomic sequencing could provide a more comprehensive view of the bacterial community and resistome composition in these samples, and this will be a focus of our future work. Finally, monthly sampling intervals may have missed short-term fluctuations in bacterial abundance or horizontal gene transfer events.

## 5. Conclusions

In summary, this study demonstrates that hospital and municipal wastewater function as interconnected reservoirs for antibiotic-resistant bacteria and plasmids of high clinical relevance. By integrating a culture-based approach with whole-genome sequencing and comparative genomics analysis, we found that environmental and healthcare niches within a single metropolitan area shared overlapping pools of strains, plasmids, and antimicrobial resistance genes. These findings underscore the importance of wastewater genomic surveillance as a complementary and non-invasive tool for tracking high-risk resistance determinants and guiding infection-prevention strategies. Integrating environmental and clinical genomic data in real time will be essential for the early detection of emerging resistance threats and for developing targeted interventions to curb their dissemination. The information we generated could also be used to inform modeling efforts to better monitor bacterial dynamics in wastewater. Ultimately, linking environmental and clinical surveillance data could strengthen antimicrobial stewardship efforts, aid responses to outbreaks of antibiotic-resistant bacteria, and inform public health policies aimed at containing the spread of antibiotic resistance.

## Data Availability

Raw Illumina sequencing reads and plasmid sequences are uploaded to NCBI Sequence Read Archive (SRA) and GenBank under BioProject PRJNA1311597. All other data is provided in the Supplemental Material accompanying the manuscript.

## Supplementary Materials

The following supporting information can be downloaded at: https://www.mdpi.com/article/10.3390/microorganisms14050961/s1, Table S1. Bacterial counts (CFU/mL) in wastewater across selective media and timepoints. Table S2. Species identification by 16S rRNA sequencing. Table S3. Antimicrobial resistance genes detected in sequenced wastewater isolates. Table S4. Circular plasmids identified from hybrid genome assemblies of wastewater isolates. Figure S1. Quantification of resistant organism burdens from hospital and municipal wastewater. Plots display colony-forming units (CFU) per mL of wastewater sourced from four different locations. Selective media included: (A) MacConkey agar (MAC) containing 1 µg/mL ciprofloxacin (CIP), (B) MAC containing 1 µg/mL cefotaxime (CTX), (C) MAC containing 1 µg/mL meropenem (MEM), (D) Pseudomonas isolation agar (PIA) containing 1 µg/mL MEM, (E) Mannitol salt agar (MSA) containing 4 µg/mL oxacillin (OXA), and (F) Bile esculin azide agar (BEA) containing 10 µg/mL vancomycin (VAN). Pairwise comparisons between CFU/mL at each location were performed via Mann–Whitney test adjusted for multiple comparisons (α < 0.0083); *p* < 0.0001: ****, *p* = 0.0005: ***, *p* = 0.0036: **.
