# Supplementary material for: Genomic Epidemiology of Antibiotic-Resistant Bacteria Sampled from Metropolitan Wastewater
Source: Microorganisms. 2026 Apr 24;14(5):961. doi: 10.3390/microorganisms14050961 (PMC13209850; doi:10.3390/microorganisms14050961)
Supplement: Supplementary file 1 [file microorganisms-14-00961-s001.zip › WW_SuppFigures.pdf]

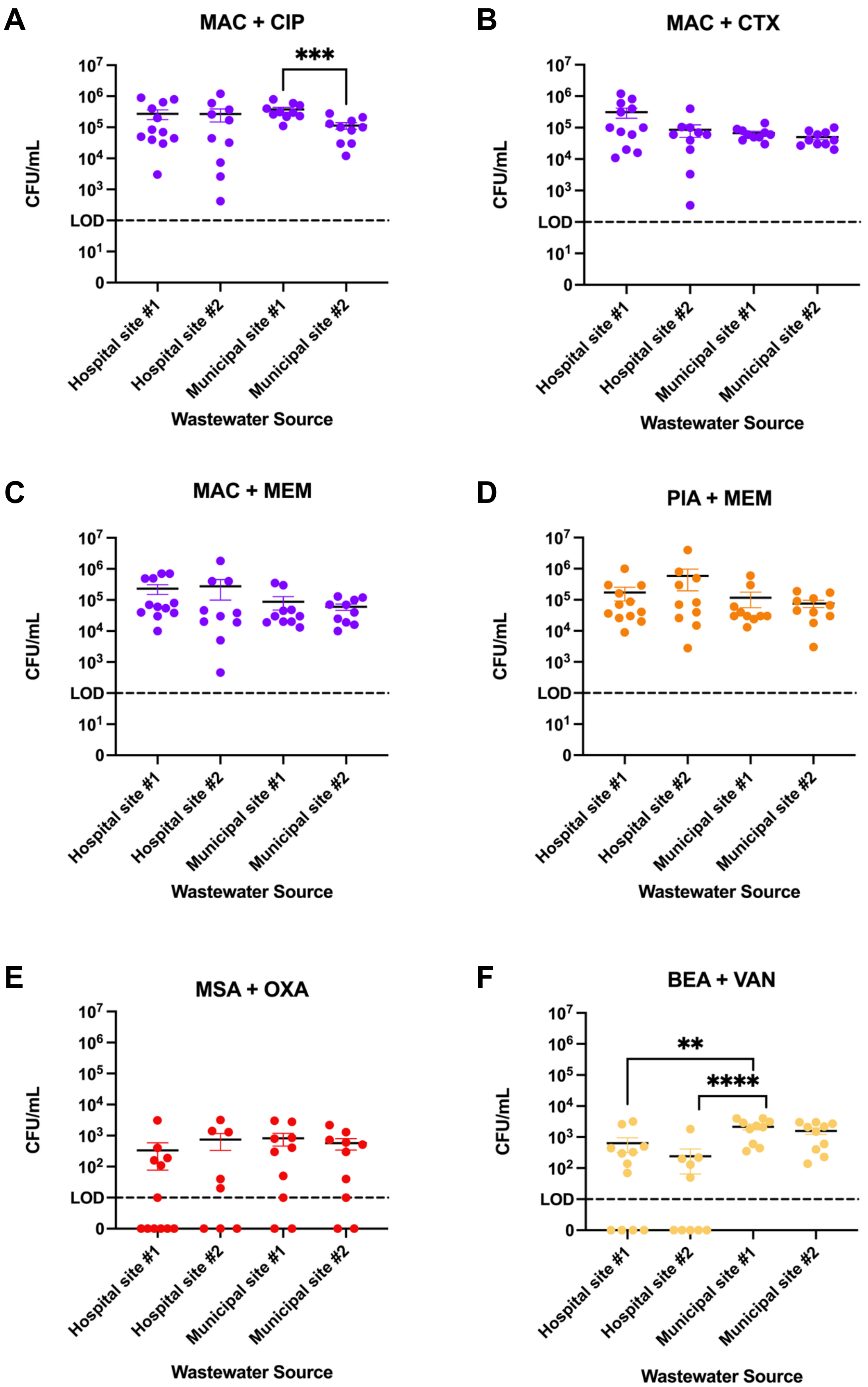

Figure S1: Quantification of resistant organism burdens from hospital and municipal wastewater. Plots display colony-forming units (CFU) per mL of wastewater sourced from four different locations. Selective medias included: A) MacConkey agar (MAC) containing 1 µg/mL ciprofloxacin (CIP), B) MAC containing 1 µg/mL cefotaxime (CTX), C) MAC containing 1 µg/mL meropenem (MEM), D) Pseudomonas isolation agar (PIA) containing 1 µg/mL MEM, E) Mannitol salt agar (MSA) containing 4 µg/mL oxacillin (OXA), and F) Bile esculin azide agar (BEA) containing 10ug/mL vancomycin (VAN). Pairwise comparisons between CFU/mL at each location were performed via Mann-Whitney test adjusted for multiple comparisons ($\alpha < 0.0083$); $p < 0.0001$: ****, $p = 0.0005$: ***, $p = 0.0036$: **.
